# ChREBP Reciprocally Regulates Liver and Plasma Triacylglycerol Levels in Different Manners

**DOI:** 10.3390/nu10111699

**Published:** 2018-11-07

**Authors:** Katsumi Iizuka, Ken Takao, Takehiro Kato, Yukio Horikawa, Jun Takeda

**Affiliations:** 1 Department of Diabetes and Endocrinology, Graduate School of Medicine, Gifu University, Gifu 501-1194, Japan; lamgerrpard@yahoo.co.jp (K.T.); bado_aberu@yahoo.co.jp (T.K.); yhorikaw@gifu-u.ac.jp (Y.H.); jtakeda@gifu-u.ac.jp (J.T.); 2 Gifu University Hospital Center for Nutritional Support and Infection Control, Gifu 501-1194, Japan; 3 Koseikai Takeda Hospital, Shimogyo-ku, Kyoto 600-8558, Japan

**Keywords:** *Chrebp*, carbohydrate response element-binding protein, fatty liver, *Fgf21*, fibroblast growth factor 21, *Angptl3*, angiopoietin-like 3, *Angptl8*, angiopoietin-like 8

## Abstract

Carbohydrate response element-binding protein (ChREBP) has an important role in the carbohydrate-mediated regulation of hepatic de novo lipogenesis, but the mechanism for how it regulates plasma triacylglycerol (TAG) levels has not been established. This study aimed to clarify the role of ChREBP in regulation of plasma TAG levels. We analyzed the metabolic changes in mice infected with an adenovirus expressing ChREBP Δ196 (Ad-ChREBP). Compared with adenovirus harboring green fluorescent protein infected mice, Ad-ChREBP-infected mice had higher plasma free fatty acid levels and paradoxically lower plasma 3-hydroxybutyrate levels through decreased fatty acid oxidation, rather than ketogenesis. Consistent with their hepatomegaly and increased lipogenic gene expression, the liver TAG contents were much higher. Regarding lipid composition, C16:0 was much lower and C18:1n-9 was much higher, compatible with increased stearoyl CoA desaturase-1 and ELOVL fatty acid elongase 6 expression. Furthermore, Ad-ChREBP-infected mice had decreased plasma TAG and very low density lipoprotein (VLDL)-TAG levels, consistent with decreased Angiopoietin-like protein 3 (Angptl3) and increased fibroblast growth factor (Fgf21) mRNA and protein levels. Finally, Ad-ChREBP infection increased white adipose tissue *Ucp1* mRNA levels with increased plasma Fgf21 levels. Because Fgf21 and Angptl3 are known to activate and suppress lipolysis in adipose tissues and oxidative tissues, ChREBP appears to regulate plasma TAG levels by modulating Fgf21 and Angptl3 levels. Thus, ChREBP overexpression led to dissociation of hepatic steatosis from hyperlipidemia.

## 1. Introduction

Excess dietary carbohydrate and fat intakes occasionally cause obesity, glucose intolerance, and dyslipidemia [1,2]. These metabolic disorders are associated with non-alcoholic fatty liver disease (NAFLD) [3,4,5]. NAFLD is characterized by excess triglyceride accumulation in the liver. The size of the hepatic triacylglycerol (TAG) pool is determined by several pathways [1]: (1) free fatty acid (FFA) supply from peripheral adipose tissues; (2) intestinal absorption from dietary fatty acids; (3) de novo lipogenesis; (4) fatty acid oxidation; and (5) very low density lipoprotein (VLDL) secretion.

De novo lipogenesis plays an important role in the regulation of hepatic lipid contents [5]. In a previous study, de novo lipogenesis was two-fold higher in subjects with NAFLD than in subjects without NAFLD [5]. De novo lipogenesis is a process that converts excess carbohydrate into fatty acids for storage [6]. Insulin and glucose induce expression of genes related to de novo lipogenesis [6]. Insulin stimulates sterol regulatory element-binding protein 1c (SREBP1c) and glucose stimulates carbohydrate response element-binding protein (ChREBP) [6]. Both of these transcription factors regulate the expression of common lipogenic genes, fatty acid synthase (*Fasn*) and acetyl CoA carboxylase 1 (*Acc1*), in the liver and consequently, de novo lipogenesis [6]. ChREBP is induced by a high-carbohydrate diet and activated ChREBP induces lipogenic gene expression [7,8,9,10]. Moreover, regulation of lipid composition plays an important role in glucose and lipid metabolism [11,12]. *Elovl6* and *Scd1* are also regulated by SREBP1c and ChREBP [13,14,15,16]. Thus, ChREBP has important roles in regulation of de novo lipogenesis and lipid composition.

Hyperlipidemia is controlled by several factors, including hepatic lipid synthesis, plasma lipid secretion, and plasma lipid clearance [17]. For example, hereditary hypertriglyceridemia is caused by decreased lipid clearance through defective lipoprotein lipase activity [18]. Although ChREBP has an important role in the regulation of liver de novo lipogenesis and lipid secretion, the relationship between ChREBP and plasma lipid clearance has not been established. Several secretory proteins are known to regulate plasma triglyceride levels. Fibroblast growth factor-21 (Fgf21) regulates plasma triglyceride levels through decreased VLDL secretion in the liver and increased TAG disposal in adipose tissues [19]. Angiopoietin-like protein 3 (Angptl3) and Angiopoietin-like protein 8 (Angptl8) can regulate plasma TAG levels through inhibition of lipoprotein lipase [20,21]. Because *Fgf21* is a target gene for ChREBP [22], we hypothesized that ChREBP may regulate plasma TAG levels by modulating secretory protein levels.

In the present study, we examined the effect of ChREBP on lipid metabolism with special reference to plasma triglyceride metabolism. Clarification of the role of ChREBP in lipid metabolism will be useful for therapeutic strategies in the treatment of non-alcoholic fatty liver disease (NAFLD) and hyperlipidemia.

## 2. Materials and Methods

### 2.1. Establishment of Mice Infected with an Adenovirus Harboring with ChREBP Δ196

Animal experiments were carried out in accordance with the National Institutes of Health Guide for the Care and Use of Laboratory Animals (NIH Publication No. 8023, revised 1978). All animal care was approved by the Animal Care Committee of the University of Gifu (No. 25-16, approval date: 2 May 2013; No. 22-26, approval date: 8 November 2010). Mice were housed at 23 °C on a 12-h/12-h light/dark cycle. At 8–9 weeks of age, male C57BL/6J mice (*n* = 6 per group) were intravenously infected with an adenovirus harboring ChREBP lacking the N-terminal 196 amino acids (ChREBP Δ196) or enhanced green fluorescent protein (GFP) as a control [23,24]. ChREBP Δ196 lacks two nuclear export signals and mimics ChREBP-β [25,26]. Mice had free access to water and an autoclaved CE-2 diet (25.5% protein, 4.6% fat, 48.9% carbohydrate; CLEA Japan, Tokyo, Japan). Mice were euthanized after 5 days by cervical dislocation. All tissue samples were immediately frozen in liquid nitrogen and stored at −80 °C until further analysis for hepatic TAG and cholesterol contents or quantitative polymerase chain reaction (PCR).

### 2.2. Liver Metabolites and Plasma Profile Measurements

Liver glucose-6-phosphate (G6P) and glycogen contents were measured as described [16,23]. Liver lipids were extracted by the Bligh and Dyer method [27] and measured using triglyceride and cholesterol E-tests (Wako Pure Chemicals, Osaka, Japan). Blood plasma was collected from the retro-orbital venous plexus after an 18-h fast (for glucose, insulin, FFA, and 3-hydroxybutyrate (OHBA)) or 6-h fast (for TAG and cholesterol). Blood glucose and OHBA levels were measured using a Free Style Freedom monitoring system (Nipro, Osaka, Japan). Plasma insulin, FFA, Fgf21, Angptl3, Angptl8, triglyceride, and total cholesterol levels were determined using commercial assay kits: mouse insulin enzyme-linked immunosorbent assay (ELISA) (H type) (Shibayagi, Gunma, Japan), NEFA C-test (Wako Pure Chemicals), mouse/rat FGF21 ELISA (R&D Systems, Minneapolis, MN, USA), Angptl3 ELISA kit (Thermo Fisher Scientific, Middletown, VA, USA), Angptl8 ELISA kit (Cloud-Clone Corp., Wuhan, China), triglyceride E-test, and cholesterol E-test, respectively. The TAG contents and cholesterol levels in lipoprotein fractions and the VLDL particle numbers were analyzed by Skylight Biotech Inc. (Akita, Japan) using gel-permeation high-performance liquid chromatography as described [28]. For serum lipoprotein analysis, serum samples obtained from six mice after a 6-h fast were pooled and measured. Lipid composition assays using gas chromatography analysis methods after methanolysis were performed by Toray Research Center (Tokyo, Japan). For hepatic lipid composition analysis, liver samples obtained from six mice after a 6-h fast were pooled and measured.

### 2.3. RNA Isolation and Quantitative Real-Time PCR

Total RNA isolation, cDNA synthesis, and real-time PCR analysis were performed as described [22,23,29,30,31]. Equal amounts of RNA from six mice were pooled and cDNA was synthesized. Real-time PCR amplifications were performed in triplicate. The relative amounts of mRNA were calculated by the comparative Ct method. *Pol2* expression was evaluated as an internal control.

### 2.4. Statistical Analysis

All values are presented as mean ± standard deviation. Data were analyzed by Student’s *t*-test. Values of *p* < 0.05 were considered statistically significant.

## 3. Results

### 3.1. Adenoviral Overexpression of ChREBP Causes Hepatomegaly without Obesity

**Figure 1:**
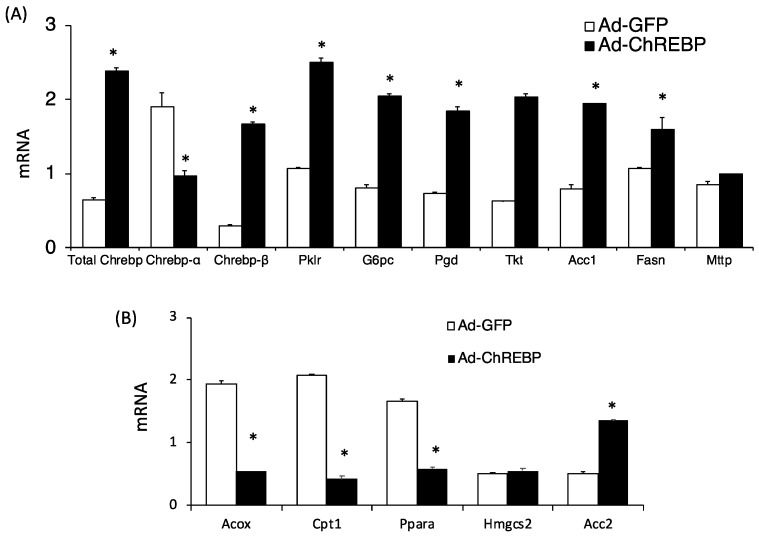
Hepatic mRNA analysis in Ad-GFP-infected and Ad-ChREBP-infected mice. The phenotypes of Ad-ChREBP-infected mice were evaluated. Male C57BL/6J mice at 8 weeks of age were infected with an adenovirus expressing ChREBP Δ196 or GFP. The mice were examined and analyzed after 5 days. (**A**) ChREBP (total *Chrebp*, *Chrebp-α*, *Chrebp-β*) and ChREBP target genes (*Pklr*, *G6pc*, *Pgd*, *Tkt*, *Acc1*, *Fasn*, *Mttp*). (**B**) Genes related to fatty acid oxidation and ketogenesis (*Acox*, *Cpt1*, *Ppara*, *Hmgcs2*, *Acc2*). Equal amounts of RNA from 6 mice were pooled, and cDNA was synthesized. RT-PCR analyses were performed in triplicate. *Pol2* expression was evaluated as an internal control. Data represent means ± SD (*n* = 3 per group). * *p* < 0.05.

We examined the metabolic effects of ChREBP overexpression in mice fed a normal chow diet. Initially, total *Chrebp* mRNA levels in Ad-ChREBP-infected mice were increased by about four times compared with those in Ad-GFP-infected mice (Figure 1A). Endogenous mouse *Chrebp-β* mRNA was similarly increased. In contrast, modest decreases were observed for endogenous mouse *Chrebp-α* (Figure 1A). ChREBP target genes (*Pklr*, *G6pc*, *Pgd*, *Tkt*, *Acc1*, and *Fasn*) were increased by ChREBP overexpression. Mttp expression was only modestly increased. Thus, Ad-ChREBP successfully infected the liver of C57BL/6J mice (Figure 1A).

Regarding phenotypes, Ad-ChREBP-infected mice showed severe hepatomegaly without body weight gain, adiposity, and increased food intake (Table 1). Plasma glucose levels were lower and plasma FFA levels were higher compared with those in Ad-GFP-infected mice. Plasma OHBA levels were much lower despite the decreased plasma glucose levels (Table 1). Plasma ketone levels are regulated by acetyl CoA supply from fatty acid oxidation, ketogenesis, and use of ketones in peripheral tissues [32]. Regarding ketogenic gene expression, Hmgcs2 levels were similar, while genes related to fatty acid oxidation were altered to promote decreased fatty acid oxidation that supplies acetyl CoA as a source of ketone bodies (Figure 1B).

Liver G6P and glycogen contents in Ad-ChREBP-infected mice were much lower than those in Ad-GFP-infected mice (Table 1). Compatible with these changes, *G6pc* mRNA levels were higher in Ad-ChREBP-infected mice (Figure 1A). Plasma triglyceride levels and cholesterol levels were both significantly lower, while liver lipid contents differed. Liver cholesterol levels were similar in the two groups, while liver triglyceride contents were much higher because of increased *Fasn* and *Acc* expression (Table 1). Thus, Ad-ChREBP-infected mice showed altered lipid metabolism compared with Ad-GFP-infected mice.

### 3.2. Adenoviral ChREBP Overexpression Causes Higher Triglyceride Contents with Altered Lipid Composition

**Figure 2:**
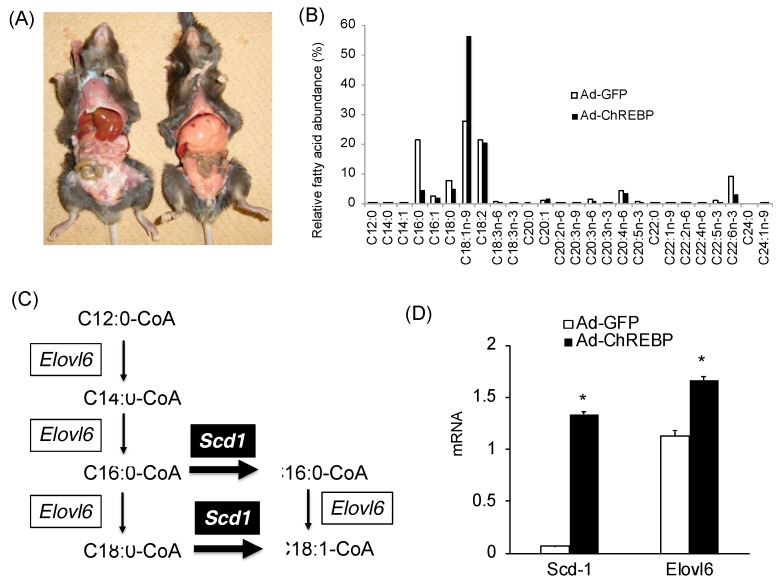
Effects of ChREBP on the liver lipid composition. (**A**) Gross anatomical views of representative mice. Hepatomegaly with fatty liver was observed in Ad-ChREBP-infected mice. (**B**) Liver lipid composition. Data are representative of six pooled samples. (**C**) *Scd1* and *Elovl6* are involved in the fatty acid elongation and lipid unsaturation pathway, respectively. (**D**) Hepatic mRNA levels of stearoyl CoA desaturase-1 (*Scd1*) and ELOVL fatty acid elongase 6 (*Elovl6*). Data represent means ± SD (*n* = 3 per group). * *p* < 0.05 vs. Ad-GFP.

Histological analyses were not performed. However, the gross anatomical views revealed hepatomegaly with fatty liver (Figure 2A). Liver triglyceride contents in Ad-ChREBP-infected mice were about seven times higher, as shown in Table 1. Regarding fatty acid composition in Ad-ChREBP-infected mice, saturated fatty acids (C16:0 and C18:0) and a monounsaturated fatty acid (C16:1) were decreased, while C18:1n-9 (oleic acid) and C20:1 were increased (Figure 2B). C20:3n-6, C20:4n-6, and C22:6n-3 were decreased. C16 acyl CoA levels were lower in Ad-ChREBP-infected mice, while C18 acyl CoA levels were much higher (Figure 2B). Consistent with these findings, mRNA levels of ELOVL fatty acid elongase 6 (*Elovl6*), a fatty acid-elongating enzyme, were increased in Ad-ChREBP-infected mice (Figure 2C,D). Furthermore, consistent with the increased C18:1n-9 levels, mRNA levels of stearoyl CoA desaturase 1 (*Scd1*) were increased in Ad-ChREBP-infected mice (Figure 2C,D).

### 3.3. ChREBP Overexpression Lowers Plasma Triglyceride Levels by Modulating Angptl3 and Fgf21 Levels

**Figure 3:**
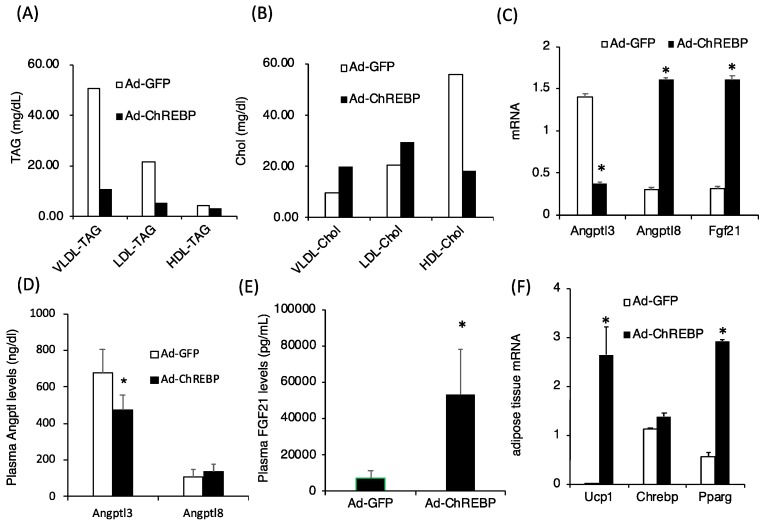
Effects of ChREBP on plasma lipid profiles and secretory proteins. (**A**) Plasma very low-density lipoprotein (VLDL)-triacylglycerol (TAG), low-density lipoprotein (LDL)-TAG, and high-density lipoprotein (HDL)-TAG levels. (**B**) Plasma VLDL-cholesterol (VLDL-Chol), LDL-cholesterol (LDL-Chol), and HDL-cholesterol (HDL-Chol) levels. Data are representative of pooled samples from six mice. (**C**) Hepatic mRNA levels of angiopoietin-like protein (*Angptl*) 3, *Angptl8*, and *fibroblast growth factor-21 (Fgf21)*. Data represent means ± SD (*n* = three per group). * *p* < 0.05 vs. Ad-GFP. (**D**) Plasma *Angptl3* and *Angptl8* levels. Data represent means ± SD (*n* = six per group). * *p* < 0.05 vs. Ad-GFP. (**E**) Plasma Fgf21 levels. Data represent means ± SD (*n* = six per group). * *p* < 0.05 vs. Ad-GFP. (**F**) mRNA levels of uncoupling protein 1 (*Ucp1*) and peroxisome proliferator-activating protein gamma (*Pparg*). Data represent means ± SD (*n* = three per group). * *p* < 0.05 vs. Ad-GFP.

The lipid profiles, plasma triglyceride, and plasma cholesterol levels were significantly lower in Ad-ChREBP-infected mice (Table 1). Regarding the plasma lipoprotein profiles, plasma VLDL-TAG, LDL-TAG, and HDL-TAG levels were much lower in Ad-ChREBP-infected mice (Figure 3A). Plasma VLDL-Chol and LDL-Chol levels were relatively higher and HDL-Chol levels were relatively lower in Ad-ChREBP-infected mice (Figure 3B). Angptl3 and Angptl8 are known to regulate lipoprotein lipase activity. *Angptl3* mRNA levels were decreased, while *Angptl8* mRNA levels were increased (Figure 3C). Regarding protein levels, plasma Angptl3 levels were significantly decreased, and plasma Angptl8 levels were slightly increased (Figure 3D). Fgf21 is known to regulate plasma lipid levels. Fgf21 mRNA and protein levels in Ad-ChREBP infected mice were much higher than those in Ad-GFP infected mice (Figure 3E). Compatible with the increased plasma Fgf21 levels, mRNA levels of uncoupling protein 1 (*Ucp1*), specific to brown adipose tissue, and peroxisome proliferator-activating protein gamma (*Pparg)* were increased (Figure 3F). ChREBP mRNA levels in adipose tissues were not affected by Ad-ChREBP infection (Figure 3F).

## 4. Discussion

**Figure 4:**
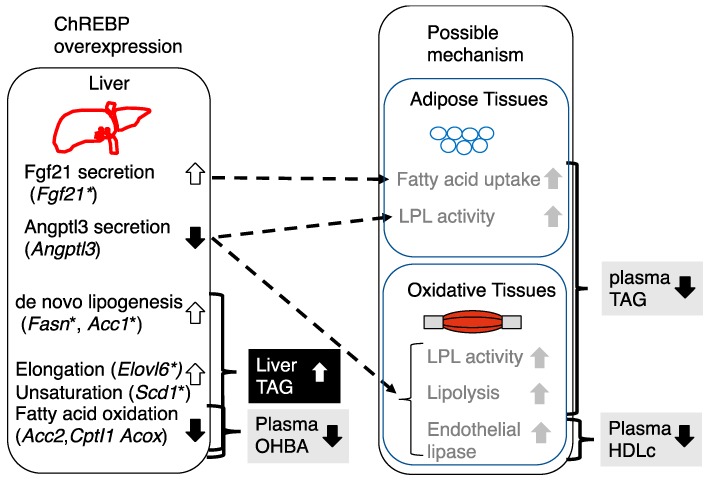
Schematic representation of the phenotypic changes in Ad-ChREBP-infected mice and possible mechanism of how ChREBP overexpression impacts liver and plasma lipid metabolism. A high-sucrose diet or high-fructose diet feeding is known to activate liver ChREBP transcription activity [7,8,9,10]. ChREBP promotes the following effects: (1) lowering of plasma 3-hydroxybutyrate (OHBA) through decreased fatty acid oxidation; (2) increase in liver triglyceride contents through increased de novo lipogenesis, fatty acid elongation and unsaturation, and decreased fatty acid oxidation; (3) lowering of plasma triglyceride; (4) lowering of plasma HDL cholesterol (HDLc). Fgf21 is known to lower plasma TAG levels by increasing TAG disposal. Angptl3 is known to inhibit lipoprotein lipase (LPL) and endothelial lipase and thereby inhibition of Angptl3 lowers plasma TAG and HDLc levels. * represented as ChREBP-target gene.

In this study, we have clarified the role of hepatic ChREBP in glucose and lipid metabolism. Consistent with a previous paper [33], we confirmed that ChREBP overexpression increased hepatic triglyceride contents, altered hepatic lipid composition, and lowered plasma glucose levels. Moreover, we observed the following effects: (1) ChREBP overexpression significantly lowered plasma ketone levels through decreased fatty acid oxidation gene expressions, and (2) ChREBP overexpression significantly lowered plasma TAG levels through altered plasma *Angptl3* and *Fgf21* levels, associated with increased WAT *Ucp1* mRNA levels. These findings suggest that ChREBP may reciprocally regulate liver and plasma triglyceride levels (Figure 4). High carbohydrate diets such as high fructose and sucrose diets are known to promote hepatic ChREBP transcription activity [7,8,9,10]. Thus, ChREBP overexpression leads to dissociation of hepatic steatosis from hyperlipidemia.

Plasma OHBA levels in Ad-ChREBP-infected mice were much lower than those in Ad-GFP-infected mice, while plasma FFA levels were much higher. Plasma ketone levels are regulated by acetyl CoA supply from fatty acid oxidation, ketogenesis, and use of ketones in peripheral tissues [32]. Regarding ketogenesis, the levels of *Hmgcs2*, encoding a rate-limiting enzyme in the ketogenic pathway [32], were similar. In contrast, for fatty acid oxidation, *Acox* and *Cpt1* mRNA levels in the fatty acid oxidation pathway were decreased, compatible with a previous paper [33]. Moreover, levels of Acc2 mRNA, which suppress CPT1 activity through mitochondrial malonyl CoA production [34], were increased. Acetyl CoA is used for glycogenosis (or gluconeogenesis) and de novo lipogenesis. Although we did not measure the hepatic acetyl CoA contents, much of the acetyl CoA pool may be used to meet the increased demand for de novo lipogenesis upon ChREBP overexpression. Therefore, the lower plasma OHBA levels in Ad-ChREBP-infected mice may arise through decreased fatty acid oxidation, rather than ketogenesis. Interestingly, ChREBP^−/−^ mice also showed lower plasma OHBA levels through decreased fatty acid oxidation, decreased supply of acetyl CoA because of lower plasma FFA levels, and lower cytosolic NAD-to-NADH ratios [31,35,36]. Although it appears contradictory that ChREBP overexpression and deletion lead to decreases in ketogenesis by decreasing β-oxidation, these findings are consistent with in vivo evidence that both *Chrebp* gene deletion and *Chrebp* gene activation fail to prevent the development of high-fat diet-induced fatty liver [31,33]. Therefore, ChREBP activity plays some roles in the regulation of ketogenesis.

In Ad-ChREBP-infected mice, both hepatic G6P and glycogen contents were decreased. G6P is one of the candidate mediators for glucose signals that activate ChREBP transcriptional activity [37]. Hepatic G6P levels are correlated with hepatic glycogen contents [38]. ChREBP knockout mice had higher hepatic G6P and glycogen contents than wild-type mice [11,36]. In the present study, hepatic G6P and glycogen contents were reduced through increased *G6pc* mRNA levels in Ad-ChREBP-infected mice. *G6pc* is a target gene for ChREBP [36,39]. Thus, ChREBP negatively regulates liver glycogen contents.

In Ad-ChREBP-infected mice, hepatomegaly caused by fatty liver was seen. These effects arise through increased gene expression related to lipogenesis. Moreover, pentose phosphate shunt pathway genes (*Pgd*, *Tkt*), which produce cytosolic NADPH for lipogenesis, were increased in Ad-ChREBP-infected mice. These findings were compatible with previous papers describing findings in ChREBP^−/−^ mice [33,36].

Fatty acid elongation and unsaturation are important processes for TAG synthesis [11,12]. Regarding fatty acid composition, the amounts of C18:1n-9 (oleic acid) were much higher in Ad-ChREBP-infected mice compared with Ad-GFP-infected mice. In contrast, the amounts of palmitic acid (C16:0) were much lower in Ad ChREBP-infected mice. These findings were compatible with those in a previous paper [33]. Elovl6 is known to elongate the carboxyl chain of fatty acyl CoA [12,15] and is regulated by not only SREBP, but also ChREBP [13]. *Elovl6* mRNA was increased in Ad-ChREBP-infected mice and thus the amounts of C18 were higher than the amounts of C16. Moreover, ChREBP regulates *Scd1* mRNA expression [16]. Scd1 catalyzes the rate-limiting step in the formation of monounsaturated fatty acids, specifically oleate and palmitoleate, from stearoyl-CoA and palmitoyl-CoA [11]. Consistent with a previous paper [33], *Scd1* mRNA was increased in Ad-ChREBP-infected mice. Unlike other unsaturated fatty acids, oleic acid cannot suppress ChREBP transcriptional activities [40]. This may also contribute to increased liver TAG storage. Consequently, the amounts of oleic acid in Ad-ChREBP-infected mice were much higher due to increased *Elovl6* and *Scd1* expression.

Regarding the physiological roles of oleic acid, oleic acid is more suitable for lipid storage than palmitic acid because of its lower melting point [41]. Moreover, palmitic acid is incorporated into DAG and consequently activates the proinflammatory PKCu–NF-κB pathway, while oleic acid is incorporated into TAG [42]. Therefore, palmitic acid is known to cause insulin resistance by attenuating insulin signaling, while oleic acid has a protective effect against insulin resistance and type 2 diabetes mellitus [42]. Compatible with these observations, our findings and previous results [33] showed that Ad-ChREBP-infected mice had lower plasma glucose levels or improved insulin resistance. Therefore, ChREBP promotes TAG storage in the liver without ameliorating plasma glucose by converting a palmitic acid into oleic acid.

In Ad-ChREBP-infected mice, plasma TAG, VLDL-TAG, LDL-TAG, and HDL-TAG levels were decreased compared with Ad-GFP-infected mice. Plasma TAG levels are regulated by de novo lipogenesis, plasma lipid secretion, and peripheral lipolysis [17]. As de novo lipogenesis and hepatic lipid secretion were rather increased due to increased gene expression (*Fasn*, *Acc1*, *Mttp*), we hypothesized that peripheral lipolysis may be increased by secretory factors that promote lipolysis. Angptl3 is known to increase lipolysis by inhibiting lipoprotein lipase [20] and Fgf21 is known to promote TAG disposal in adipose tissues [19]. In Ad-ChREBP-infected mice, hepatic *Angptl3* mRNA and plasma Angptl3 protein levels were decreased, while hepatic *Fgf21* mRNA and plasma Fgf21 protein levels were significantly increased. In support of these findings, Fgf21 is known to induce adipose tissue *Ucp1* and *Pparg* mRNA expression [43,44]. Interestingly, *Ucp1* and *Pparg* mRNA expression were induced in white adipose tissue. Taken together with the finding that Ad-ChREBP was not present in white adipose tissue, these observations suggest that increased plasma Fgf21 levels could modulate adipose tissue function in Ad-ChREBP-infected mice. The findings suggest that decreased Angptl3 and increased Fgf21 may promote peripheral TAG disposal. Angptl8 is known to increase plasma TAG levels by suppressing lipoprotein lipase [21] and *Angptl8* is a target gene for ChREBP [45]. Although *Angptl8* mRNA levels were increased, plasma Angptl8 protein levels were not significantly increased. In Ad-ChREBP-infected mice, only *Angptl8* mRNA levels were significantly increased. Moreover, in *Angptl*3^−/−^ mice, Angptl8 administration did not increase plasma triglyceride levels [21,46]. These observations suggest that Angptl3, rather than Angptl8, may contribute to the lower plasma TAG levels in Ad-ChREBP-infected mice.

The mechanism for how ChREBP regulates Angptl3 expression has not been established and further investigations are needed. Meanwhile, plasma cholesterol levels in Ad-ChREBP infected mice were lower. VLDL-Chol and LDL-Chol levels were higher and HDL-Chol levels were lower. Regarding plasma HDL-Chol levels, lower Angptl3 mRNA levels lead to lower plasma HDL-Chol levels [47]. In particular, Angptl3 inhibits the phospholipase activity of endothelial lipase, and lowering Angptl3 may thereby decrease plasma HDL-Chol levels [48]. These observations are compatible with the findings in our Ad-ChREBP-infected mice. Thus, ChREBP may regulate plasma lipid levels partly through FGF21 and Angptl3.

## 5. Conclusions

We clarified that (1) ChREBP negatively regulates plasma ketone levels, and (2) ChREBP differently regulates hepatic and plasma triglyceride levels. Under normal conditions, excess intake of carbohydrates such as fructose or sucrose is converted into triglyceride through glucose-activated ChREBP. ChREBP promotes hepatic de novo lipogenesis by induction of lipogenic gene expression. ChREBP lowers plasma ketone levels by inhibiting fatty acid oxidation. Furthermore, ChREBP lowers plasma TAG levels through TAG disposal in adipose tissue and oxidative tissues by decreasing plasma Angptl3 levels and increasing plasma Fgf21 levels. Therefore, the discrepancies between plasma and liver TAG levels in Ad-ChREBP mice revealed that ChREBP reciprocally regulates hepatic and plasma TAG levels in different manners. With the intake of a high-fructose or high-sucrose diet, Fgf21 and Angptl3, rather than Angptl8, may be better targets for the improvement of hypertriglyceridemia.

## Figures and Tables

**Table 1:** Phenotypic comparisons between Ad-GFP-infected and Ad-ChREBP-infected mice.

	Ad-GFP	Ad-ChREBP
BW (g)	24.35 ± 1.47	22.46 ± 0.66
Liver (%BW)	6.01 ± 0.31	9.80 ± 1.69
WAT (%BW)	1.38 ± 0.16	1.09 ± 0.45
BAT (%BW)	0.56 ± 0.36	0.48 ± 0.10
Food intake (g)	4.03 ± 0.58	3.46 ± 0.56
Plasma glucose (mg/dl)	110.7 ± 25.5	46.7 ± 13.5 *
Plasma OHBA (mM)	1.28 ± 0.15	0.61 ± 0.36 *
Plasma FFA (mM)	0.56 ± 0.22	1.35 ± 0.65 *
Plasma Insulin (ng/mL)	0.32 ± 0.16	0.43 ± 0.25
Plasma total cholesterol (mg/dl)	76.21 ± 9.23	59.36 ± 13.01 *
Plasma triglyceride (mg/dl)	92.94 ± 28.54	65.91 ± 25.54 *
Liver glycogen (mg/g liver)	112.6 ± 38.1	20.3 ± 3.3 *
Liver glucose-6-phosphate (μmoles/g liver)	0.41 ± 0.06	0.19 ± 0.05 *
Liver total cholesterol (mg/g liver)	1.21 ± 0.25	1.61 ± 0.45
Liver triglyceride (mg/g liver)	10.5 ± 3.5	73.8 ± 37.9 *

Abbreviations: BW, body weight; WAT, white adipose tissue; BAT, brown adipose tissue; OHBA, 3-hydroxybutyrate; FFA, free fatty acid. Data represent means ± SD (*n* = 6 per group). * *p* < 0.05.
